# New Developments in Emotion-Focused Therapy for Social Anxiety Disorder

**DOI:** 10.3390/jcm9092918

**Published:** 2020-09-10

**Authors:** Ben Shahar

**Affiliations:** The Paul Baerwald School of Social Work and Social Welfare, Hebrew University, Jerusalem 91905, Israel; ben.shahar@mail.huji.ac.il

**Keywords:** emotion-focused therapy, anxiety disorders, social anxiety disorder, shame, self-criticism, psychotherapy, psychological treatment, emotion, trauma

## Abstract

Social anxiety disorder (SAD) is a highly complex, chronic, disabling and costly anxiety disorder. Although cognitive-behavioral therapy (CBT) is effective for many patients, many others do not respond to CBT or remain considerably symptomatic at the end of treatment. Pharmacological effects are also modest. More empirically-supported treatment options are needed in order to increase patient access to effective treatment. Emotion-focused therapy (EFT) shows great promise in treating SAD effectively and is particularly suitable for treating SAD because pervasive emotional avoidance, difficulties with emotional differentiation, and high levels of self-criticism, which are central psychopathological processes in SAD, are also primary therapeutic targets in EFT. EFT is based on the assumption that the most efficient way to change a maladaptive emotion is not through reason or skill learning, but through the activation of other, more adaptive emotions. EFT aims to access shame-based emotional memories that underlie SAD, and transform them by exposing them to new adaptive emotional experiences, such as empowering assertive anger, grief, and self-compassion. In this paper, the core features of EFT for SAD are presented, as well as the EFT view of dysfunction in SAD and EFT change processes. Research findings regarding the effectiveness of EFT for SAD are presented together with initial findings regarding mechanisms of change occurring during treatment.

## 1. Introduction

Social anxiety disorder (SAD) is characterized by persistent fears in a range of social and performance situations in which the individual might be scrutinized or judged by others [1]. Individuals with SAD are afraid that perceived flaws will be exposed and that others will criticize, mock, or dismiss them, resulting in unbearable experiences of shame and rejection. People with SAD usually experience intense anxiety before such situations and tend to avoid them, often leading to extreme impairment in multiple life domains [2,3]. SAD is associated with lower income, lower educational achievements, lower job positions, higher rates of unemployment, more difficulties initiating and maintaining romantic relationships, and more utilization of medical services [4]. These difficulties often lead to psychological comorbidities, such as depression, substance abuse, and suicide [5]. SAD usually starts in early adolescence and does not remit unless treated successfully [6,7]. It is highly prevalent, with estimated lifetime and 12-month prevalence rates of 12.1% and 7.1%, respectively [8]. Therefore, it is clear that there is a crucial need for effective psychological and pharmacological treatments for this highly complex, prevalent, chronic, and disabling anxiety disorder.

At present, treatment guidelines [9,10] recommend various forms of cognitive-behavioral therapies as front-line evidence-based treatments for SAD, such as Clark’s individual model [11,12] and Heimberg’s group therapy model [13]. Numerous randomized control trials and a few meta-analytic studies [14,15,16] have shown that cognitive-behavioral therapy (CBT) is an effective treatment for SAD, but a substantial number of patients do not respond to CBT or remain symptomatic at the end of therapy [17,18,19]. For example, in the largest randomized control trial conducted to date comparing Clark’s individual CBT to psychodynamic therapy [20,21], 40% of patients receiving CBT were classified as non-responders at the end of treatment and 30% remained non-responders after two years. In a study that computed response rates to CBT for different anxiety disorders based on randomized clinical trials conducted between 2000 and 2014, response rates for SAD were 45.3% at post-treatment and 55.5% at follow-up [22]. These studies and others show that although a majority of patients benefit from CBT, there is still considerable room for improvement. The large number of non-responders to CBT desperately calls for improving CBT as well as developing additional treatment options. Having just one evidence-based treatment option for such a prevalent, chronic, and disabling condition, especially given that most people with anxiety disorders do not end up getting evidence-based treatment [23], is problematic. In fact, only approximately one-third of individuals with SAD reported receiving treatment specifically for SAD [3]. Therefore, in order to increase access to evidence-based treatments it is crucial to have several effective, empirically-supported treatment options.

## 2. Emotion-Focused Therapy

In recent years, clinical researchers have indeed developed additional treatment approaches for SAD, such as interpersonal [24] and psychodynamic therapy [20,21]. One important recent development involves adapting and using Emotion-Focused Therapy (EFT) for SAD [25,26,27,28]. EFT is a brief (usually 16–24 sessions), experiential treatment that has developed from the humanistic tradition [29,30]. It is an empirically-supported treatment for depression [9,31,32,33] and has also shown initial promising effects in treating symptoms resulting from severe and repeated childhood abuse (complex trauma) [34,35] and generalized anxiety disorder [36]. Like CBT and psychodynamic treatments, EFT has developed as a transdiagnostic approach [37], which means that it can be adapted to be used with various forms of psychopathology.

As a transdiagnostic treatment, EFT is based on the premise that emotions are fundamentally adaptive resources in healthy human functioning [38], and that accessing, activating, and productively processing emotions are crucial for successful outcome in psychotherapy [39,40]. Based on basic emotion research [41,42], emotions are viewed as providing vital information for the individual that helps in surviving and thriving. Emotions alert us to crucial environmental cues that are relevant to our survival and well-being and organize us to respond to them effectively. For example, fear alerts us to immediate danger and organizes the body to behaviorally respond (fight, flight, or freeze) [43], anger is a response to boundary violation and organizes us to protect our boundaries, sadness is a response to loss and organizes us to reach out for comfort, disgust is a response to a noxious object or stimulus and organizes us to expel, shame is a response to being devalued and organizes us to conceal perceived flaws [44], and so on. Emotions tell us what is important to us in a given moment and whether our goals and needs are being met, and organize us to take action in order to meet our needs and goals.

One of the primary contributions of EFT to clinical research and practice is the distinction between several forms of emotional response [45]. A first distinction is between primary and secondary emotional responses. Primary emotions are immediate responses to a situation, such as fear in response to danger, sadness in response to loss, or shame in response to exposure of flaws. Secondary emotions, in contrast, are triggered in response to primary emotions, such as when people feel guilty about their primary anger, or rage in response to shame. Secondary emotions often obfuscate, conceal, or interrupt primary emotions. A second distinction is between primary adaptive and primary maladaptive emotions. Both are immediate responses to a significant trigger (danger, loss, boundary violation, devaluation). Primary adaptive emotions provide useful information and organize the individual to respond adaptively to meet important needs. For example, it is adaptive to grieve in response to a loss, to escape a life-threatening situation, or to protect one’s boundaries in the face of intrusiveness or exploitation. While primary adaptive emotions are immediate responses to current situations, primary maladaptive emotions are responses to current situations imbued in memories of past traumatic experiences. For example, when a person responds with intense shame to a relationship breakup in the present, the emotional response is often based on past shame memories of rejection or humiliation. Primary maladaptive emotions are core painful emotions that patients often attempt to avoid. They are maladaptive because they do not prepare for adaptive action to deal with the current situation.

Distinguishing among different emotional responses is crucial because each one calls for a different therapeutic intervention. Secondary emotions are usually symptomatic emotions; they require validation from the therapist and then exploration in order to access the underlying primary emotions that generate them. Usually, secondary emotions obscure primary maladaptive emotions such as shame or fear of abandonment resulting from past traumatic experiences. Primary maladaptive emotions need to be accessed and activated in therapy so that they can be exposed to primary adaptive emotions. Thus, one of the central guiding principles in EFT is that the best way to change core maladaptive emotions is not through reason or skill learning, but through the in-session activation of primary adaptive emotions [46]. Shame, for example, is transformed through its activation together with the activation of anger that promotes assertive boundary-setting, sadness that promotes grieving, and self-compassion that promotes soothing. Thus, the transformative process is primarily facilitated by new (emotional) experiences rather than through learning new skills or new insights.

A large body of literature has shown that these emotion categories can be reliably coded by independent objective raters and that they can reliably predict treatment outcomes. For example, one study found that the presence of more secondary emotions in the working phase of treatment predicted worse outcomes, and primary adaptive emotions predicted improved outcomes [47]. Furthermore, movement from secondary emotions, to primary maladaptive emotions, to primary adaptive emotions has been related to positive treatment outcomes [48,49,50,51,52,53].

In sum, the primary goal of EFT is to help patients develop a better awareness of their emotions, label them in words, learn to better regulate them, accept them, tolerate them, and make sense of them, so that the adaptive information they contain can be utilized. When emotions become maladaptive as a result of trauma (for example, when shame is internalized into a chronic sense of worthlessness as a result of childhood maltreatment [54]), the therapeutic goal is to access and activate trauma-based maladaptive emotion schematic memories in order to transform them.

## 3. An EFT View of Etiology of Social Anxiety Disorder

Distinguishing between various types of emotional responses also allows for developing emotion-based case conceptualizations and intervention maps for each patient or group of patients. Social anxiety is characterized by a set of problematic emotional responses based on shame as the core primary maladaptive emotion and several secondary emotional processes designed to cope with it [28].

From an emotion-focused perspective, social anxiety is essentially shame anxiety [26]. Socially anxious individuals are not afraid of social situations per se, they are afraid that their perceived flaws, weaknesses, or vulnerabilities might be exposed in these situations, which might lead to ridicule, contempt, and ultimately rejection or exclusion [55]. Social situations characterized by unconditional acceptance, appreciation, and complete absence of judgment from others are not likely to trigger anxiety even among the socially anxious. Thus, shame is at the heart of social anxiety, and there is a considerable amount of research showing the association between shame and SAD [56,57]. Shame is a complicated emotion without a fully agreed-upon definition. According to Sznycer’s information threat theory [44,58,59,60], shame is a self-conscious emotion (like pride and guilt) designed to solve interpersonal evolutionary-based problems related to social valuing and devaluing. Specifically, shame is triggered when the individual is socially devalued and organizes the individual to prevent negative information from being spread and to avoid further devaluation. Thus, the action tendency in shame is to hide and conceal one’s flaws in order to prevent or minimize exposure of flaws. When people experience shame they often say that they feel small or exposed, they hide their face, and often say things like “I wish the ground would swallow me up”.

Shame sets a particular mode of processing in action, efficiently orienting individuals to potential shame-related threats (e.g., negative evaluation from others) [61]. This mode of processing often involves biased attention to shame-related threats and biased interpretation of ambiguous environmental stimuli as threatening. While cognitive-behavioral models of social anxiety view these kinds of biased information processing as core etiological processes in SAD [62,63], and as primary targets for intervention, in EFT they are viewed as specific shame-related processing characteristics, derivatives of shame activation. As such, they operate primarily when shame or shame anxiety are activated as opposed to when people feel safe. Supporting this, in one study participants with SAD displayed biased attentional process (in a visual search paradigm) only under conditions of social threat. Socially anxious participants who were not induced to experience social threat did not monitor the environment hyper-vigilantly [64]. In another study [65], socially anxious individuals did not show more submissive behaviors compared to non-anxious participants in social situations where anxiety levels were low. Therefore, as described in more detail later, the therapeutic goal in EFT is not to train patients to pay attention to nonthreatening cues, as in attention bias modification training [66], nor to train them to challenge their distorted view of themselves [67] (even though these interventions have positive effects), but to fully activate the shame-based emotion scheme and its associated memories in therapy sessions in order to expose them to new emotional experiences, and in this way, to more fundamentally restructure it.

Socially anxious individuals feel inherently flawed, damaged, worthless, weak, different, or inferior, and they live in constant anxiety that this “secret” will be discovered by others. Therefore, they often use a variety of strategies to prevent being exposed as flawed and to prevent negative information about them from being spread, which to them, is the worst possible scenario. One of the central strategies for preventing flaw exposure is to have high levels of self-criticism [54,68]. Research shows that socially anxious individuals are highly self-critical [69,70]. Harsh self-criticism is designed to make sure the individual appears strong, capable, fit, and attractive and thus prevent social devaluation. It is important to view behavioral avoidance in SAD not just as a way to avoid symptomatic anxiety, but as part of a broader conceptualization involving shame and self-criticism. SAD patients avoid social situations in the context of a harsh internal critic delivering messages such as: “if you speak up in the meeting everyone will see how incompetent you are and they will finally realize you are not fit for this job”. During social situations, the internal critic often closely monitors everything the individual says or is about to say, as well as how the individual appears or performs. The goal of the self-critical, self-monitoring process is to search for potential personal flaws in speech, appearance or performance and to warn about them. As a result of this shaming self-criticism, people with SAD avoid social situations to prevent flaws from being exposed and negative information about the self from being spread. Following social situations, self-criticism is particularly strong. This is called post-event processing or post-event rumination in cognitive-behavioral models, but it is crucial to recognize that this is a self-critical process as the individual often self-attacks over perceived mistakes that might have led to exposure of flaws.

Thus, self-criticism has a central role in maintaining social anxiety. It operates before, during, and after social situations, with all three types of operations designed to minimize or prevent social risk taking, flaw exposure, and the activation of shame. At the same time, self-critical attacks are shaming in and of themselves. A self-critical remark such as “if you say something in class everyone will see how stupid you are” is protecting against flaw exposure and shaming at the same time. Thus, there is a circular process whereby attempts to regulate shame also maintain it.

In a related fashion, socially anxious people often equate emotional expression, particularly emotions that are viewed as more vulnerable (sadness, shame, fear), as a sign of weakness, and therefore avoid these emotions, which often leads to problems with emotion labeling and differentiation, together with difficulties with emotion regulation. A large body of literature shows that SAD is associated with experiential avoidance [71], difficulties with emotion differentiation (e.g., the ability to describe one’s emotions in detail) [72], poor emotion knowledge [73], and difficulties with emotion regulation [74]. A recent study found that, relative to a non-anxious control group, socially anxious participants believed that emotional experience and expression should be controlled, and that these beliefs predicted more emotional suppression on a daily basis [75]. These emotional processing difficulties are all regulatory efforts to prevent being exposed as flawed and being devalued.

Although high levels of self-criticism and other emotional processing difficulties are central maintaining processes in SAD, it is also important to understand and therapeutically address the origins of shame-proneness and self-criticism. The fact that social anxiety usually begins in early adolescence emphasizes the importance of early experiences in the etiology of the disorder. Considerable research shows that childhood maltreatment, and particularly emotional abuse and emotional neglect, are associated with shame-proneness [76] and SAD [54,77,78,79]. Such traumatic experiences lead to internalization of shame and to a chronic sense of worthlessness, inferiority, and inadequacy. This sense of inadequacy is experienced as the ultimate, most fundamental truth about the self, and SAD patients are essentially in constant anxiety that this “truth” will be discovered by other people. Again, harsh self-criticism, self-monitoring, as well as emotional and behavioral avoidance are designed to prevent this “truth” from being discovered.

## 4. The Process of Change in EFT for Social Anxiety

There are several emotional processing mechanisms in EFT for SAD. During treatment, patients learn to approach their emotions, attend to them, accept them, label them in words, and contextualize them by making sense of them as part of a larger life narrative. These processes are designed to help patients manage their emotions more flexibly and use them productively [80]. The first task in treatment is forming a warm, trusting, and secure therapeutic relationship. Bonding is crucial with every psychotherapy patient but it can be especially challenging with socially anxious patients because they tend to be extremely sensitive to criticism and invalidation. As described earlier, they tend to be hypervigilant about the possibility that others, including their therapist, will judge them and find them inadequate or flawed. Therefore, EFT therapists are highly attuned and present, creating a validating and supportive environment. Patients must feel completely and unconditionally accepted, that they can bring their authentic feelings, needs, hopes, excitements, and wishes to the encounter while deeply trusting that they will not be shamed. A secure bond with a validating, warm, compassionate, and accepting therapist facilitates exploration of vulnerable avoided emotions (which is needed for the emotional transformation process) and is also therapeutic in and of itself as it provides a corrective emotional experience [81]. Patients might internalize the validating and compassionate attitude of their therapist and thus might treat themselves with more self-compassion, which is the primary antidote for self-criticism [82].

However, the most central emotional processing mechanism is emotional transformation, involving activating shame in order to restructure it by accessing adaptive emotions that have been previously avoided. This change mechanism, called changing emotion with emotion [45,51,52], is based on the idea that the most powerful way to change maladaptive shame is by accessing and activating other, adaptive emotions.

When patients first enter therapy, shame is usually obscured by secondary emotions such as shame anxiety, general distress (hopelessness, helplessness), depressive symptoms, and substance abuse resulting from having the disorder. Some patients begin treatment in a state of under-regulation, i.e., they experience secondary emotions with high arousal and they need help down-regulating their emotions. Other patients begin therapy in a state of over-regulation, i.e., they have little access to emotions, tend to intellectualize and “tell stories”, and often have difficulties labeling their emotions and understanding them. Whatever the initial experiential state, therapists need to validate secondary processes but then to “explore through” them in order to access and activate shame. This is particularly challenging, because shame is an extremely painful and vulnerable emotion. However, once shame is activated therapists can work on restructuring it.

Shame transformation can be done using a variety of interventions, the three most central being: two-chair work for self-criticism; empty-chair work for lingering unresolved emotions toward key figures involved in the original traumatic circumstances (abusing and/or neglecting parents and other caregivers, bullying peers); and two-chair enactment for self-soothing and self-compassion. In two-chair work for self-criticism, the patient is essentially enacting the self-critical internal dialogue. The patient is asked to sit in one chair and express the harsh self-critical messages (the critic’s chair) and then to respond to these messages from another chair (called the experiencing-self chair). This intervention is based on several steps that take place over several sessions [83].

(1) Initially, in the critic’s chair, the patient expresses shame anxiety (“if you say something in a meeting, it will be a disaster, everyone will see how stupid you are”). In response, in the experiencing-self chair, the patient often feels a variety of secondary emotions such as helplessness, despair, and anxiety. Although this step is initial, it often leads to some relief and symptomatic improvement because it fosters a metacognitive understanding of the social anxiety symptoms. Patients begin to understand how the anxiety is generated, and a clearer focus for treatment emerges.
(2) Second, as the therapist helps the patient to express and even amplify specific and contemptuous messages in the critic’s chair, the emotional response in the experiencing-self chair is deepened and differentiated, and the patient begins to experience shame (“I feel small and worthless, and I want to shrink into the ground and hide”). The therapist helps the patient stay in this state and provides a great deal of validation for these painful feelings.
(3) Embedded in shame is a fundamental need to be unconditionally accepted, to belong, and to be recognized, seen, and validated. When patients allow themselves to experience shame within the therapy session, they often gain access to these shame-based adaptive needs. Therapists help patients in this process by asking them what they need, and then by helping them to clearly articulate these needs.
(4) With the help of the therapist, patients begin to express more adaptive emotions related to these needs not being met. For example, boundary-setting assertive anger toward the internal critic (“I need you to get off my back and stop monitoring everything I say”), sadness about the many losses they have experienced as a result of social anxiety (loss of relationships, career opportunities, and so on), and pride (“I have actually achieved quite a bit”). In addition, the internal critic often softens, expressing compassion toward the suffering expressed in the experiencing-self chair. Assertive anger, sadness of grieving, pride, and self-compassion are adaptive emotional experiences that help to empower the patient and to undo shame. The patient feels entitled to have these fundamental needs met, which is the opposite of feeling inadequate and undeserving.
(5) Clear articulation and expression of the critic’s function is also a crucial component of this process, which further helps to activate primary adaptive emotions. Therapists help patients, when in the critic’s chair, to describe what they are doing and why (“I am watching everything you say so that you don’t say anything stupid, so that you’re not exposed”, “my job is to make sure no one finds out the real truth about you – that you are inadequate”). These messages tend to trigger primary adaptive anger and sadness in the experiencing-self-chair (“I am tired of being oppressed like that, I need my freedom to be who I am. I am not willing to take it any more”).

A similar process takes place in empty-chair work with important figures that mistreated the patient, usually during childhood and/or adolescence. In empty-chair work, the patient enacts an imaginal dialogue with the trauma-causing individual. In this task, original shame memories usually surface and are thus open to reconsolidation [84]. For example, a patient might enact an imaginal dialogue with his father who was emotionally abusive. During the dialogue, specific shame memories might come up and activate shame in the session. The patient might then express important unmet needs (“I needed you to just accept me for who I am”), which often elicit anger for not having these needs met (“I am angry at you for humiliating me, what you did was not fair”) and sadness for the losses involved (“I could never be just an innocent happy child”). Again, adaptive sadness and anger undo the shame by connecting the patient to healthy needs for boundaries, unconditional acceptance, and support. Anger and sadness help patients feel entitled to have these needs met, which helps to counter core beliefs of inadequacy. Sometimes empty-chair work ends with holding the other accountable for the maltreatment and letting go of the hope that the other will change. At other times, resolution is achieved in a process of developing empathy for the injuring other, developing a more complex view of the other (as having both good qualities and bad qualities), and even forgiving the other [85]. Both the two-chair and the empty-chair interventions have been thoroughly studied using task analytic methods [86] among patients suffering from depression and repeated childhood maltreatment (complex trauma). For example, task analytic studies have found that softening of the critic (i.e., the process by which the internal harsh critic becomes softer and expresses compassion during the task) is a crucial ingredient in two-chair work [87,88,89]. In studies of empty-chair work, those patients who expressed anger and sadness, who articulated previously disowned unmet needs, and who experienced a shift in the view of the injuring caretaker had better outcomes [85,90].

Finally, it is crucial to directly foster self-soothing and self-compassion capacities among socially anxious patients. Growing up in abusive, neglecting, shaming or emotionally cold environments, these patients have not internalized the ability to self-soothe and self-validate in times of distress. Quite the opposite, they develop a self-critical attitude to ensure that others do not devalue them. Moreover, they often find self-compassion and compassion from others to be threatening. If one’s agenda is to avoid failure and rejection at all cost, compassion might be perceived as weak and complacent and therefore dangerous. Indeed, research has shown that socially anxious individuals have lower levels of self-compassion compared to individuals without SAD, that less self-compassion is associated with more severe SAD symptoms [91,92], and that interventions designed to increase self-compassion among socially anxious patients can have positive effects [93,94].

In EFT, it is assumed that patients internalize the compassionate, accepting and validating attitude of the therapist. However, in addition, EFT therapists often use a specific form of two-chair enactment to further activate self-compassion. In this enactment, therapists facilitate a dialogue between the adult self in one chair and the vulnerable wounded self in the opposite chair. Visualizing the wounded child is often a powerful trigger for compassion, and the therapist helps the patient to express this compassion to the wounded child. Subsequently, the patient is asked to take the child’s position in order to internalize the experience of receiving compassion. In other instances, guided imagery can be used, in which the patient is guided to enter a specific painful childhood scene (for example being bullied) and offer compassion, support, guidance, and protection to the child in the scene. Overall, these interventions help to activate compassion and soothing, which can transform shame. Nevertheless, it is crucial to reemphasize that work on developing self-compassion in EFT is not based on deliberate skill training but on a transformative process. In other words, developing self-compassion is not based on an independent set of compassion-specific techniques designed to soften one’s harsh and contemptuous inner critical voice. Rather, it is part of a larger transformational process, in which patients express boundary-setting anger toward their critical stance, and mourn the losses accrued as a result of not leaving one’s comfort zone.

### Key Differences between EFT and CBT for SAD

EFT is a new development in the SAD treatment literature. As such, it is important to note a few central points that distinguish it from CBT, especially given that current forms of CBT have recently moved away from traditional cognitive restructuring techniques and instead incorporate more experiential techniques, such as behavioral experiments, and techniques based on video feedback [95]. The ultimate goal in CBT for SAD is to identify and correct patients’ judgmental, attentional, and interpretational biases [96]. For example, one of the central interventions in Clark’s cognitive model is an experiment in which patients are asked to engage in an anxiety-provoking activity (talking to a stranger in the session for five minutes) while trying to maximize self-focused attention and safety behaviors, and then to repeat the activity while dropping self-focused attention and safety behaviors. Patients experientially learn that without self-focused attention and safety behaviors they feel less anxious and appear less anxious and are therefore encouraged to continue to drop these maladaptive behaviors at home. Similarly, in other behavioral experiments, patients test their beliefs (I am not going to have anything to say, my voice will be shaky) in activities such as group discussions and learn that their predications are inaccurate. Thus, the main goal in Clark’s cognitive model is helping patients to develop awareness of biased cognitive processes and to correct them. There is indeed a small but growing number of process studies in CBT for SAD, and most of them found that improvements in negative cognitions, threat appraisals, self-focused attention, anticipatory processing, post-event rumination, and avoidance were associated with improved outcomes [97].

In contrast, in EFT interventions are not directed at helping patients understand that their perceptions are biased or exaggerated and to correct them. In EFT, the maintaining cognitive factors that are emphasized in cognitive-behavioral models are viewed as corollaries of shame. The primary goal is not to correct those biases but to activate shame in the session (for example, as patients engage in an imaginary dialogue with an abusing figure and report feeling small, damaged, and wanting to shrink into the ground and hide). The primary goal in EFT is not merely exposure to shame so that patients realize they are able to tolerate it and survive. The ultimate goal is fundamentally restructuring shame by activating a new, adaptive, opposing, emotional state [28,51]. Thus, the process of change is based on activating new emotional experiences that transform maladaptive shame. When patients experience and express boundary-setting anger towards abusing figures (or towards their own harsh internal critics), grief for all the losses they have suffered, and compassion toward themselves, while also receiving unconditional acceptance from their therapist, they feel more empowered, more confident, and more deserving, and these processes change their shame-based beliefs. Anger, sadness, and compassion cure anxiety and shame.

## 5. Efficacy of EFT for SAD

Two studies thus far have provided evidence supporting the efficacy of EFT for SAD [27,98]. Although preliminary, both studies showed large effect sizes pointing to the potential of EFT to become an empirically-supported treatment option for SAD. In the study conducted by our group [27], 12 socially anxious patients participated in a multiple-baseline single-case design (also called staggered baseline design), in which they were randomized to wait four, eight, or 12 weeks between their initial intake interview and the beginning of treatment. Multiple baseline designs are becoming increasingly popular in psychotherapy research because they have several advantages: They can demonstrate that change has occurred, that it is significant, and that it is a result of the intervention and not of time or reactivity to multiple assessments; they have more power than wait-list design because participants serve as their own controls; and they have greater external validity than a wait-list randomized design because it simulates real-world settings in which different patients wait different amounts of time before treatment, similar to the reality in community mental health centers.

The results of this study provided initial evidence regarding the efficacy of EFT for SAD. Social anxiety symptoms, assessed with multiple measures, did not change during the baseline period (regardless of its length), significantly improved during the treatment phase, and remained improved during a 12-month follow-up phase. Effect sizes (Cohen’s *d*) for all measures were large. For example, effect size for the clinician-administered Leibowitz Social Anxiety Scale (LSAS) [99], which is considered the gold standard assessment tool in SAD randomized trials, was 2.37. Effect size for the Social Phobia Inventory (SPIN) [100] was 1.52. All patients showed reliable change [101] on the LSAS. Self-criticism and self-compassion also significantly improved as a result of treatment. The second study [98] compared the effectiveness of EFT and client-centered therapy among 52 patients suffering from SAD. In this study, effect sizes for both treatment groups were large. For example, effect sizes for the SPIN were 1.01 for the client-centered group and 1.75 for the EFT group. These effect sizes are comparable to those observed in randomized trials of CBT.

In addition, we have launched a program of research to study the change processes in EFT for SAD. Process studies (as opposed to outcome studies) are designed to investigate the complex pattern of changes occurring during treatment that eventually lead to treatment success. These studies cannot be based solely on self-report questionnaires as commonly done in cognitive-behavioral research. Instead, to gain a deeper understanding of the moment-by-moment processes that unfold during sessions, researchers need to employ careful objective observations of in-session processes and to then examine relationships between these processes and outcome. In one study [102], we found that the amount of in-session shame decreased during the treatment whereas amount of in-session assertive anger increased, consistent with the idea that experiences of adaptive anger help to transform (and reduce) shame. Additionally, when patients experienced grief in a given session it predicted less social anxiety symptoms during the following week, suggesting that experiencing grieving sadness during sessions is adaptive and therapeutic. This finding is consistent with another study, currently under review, in which we compared in-session emotions of two patients who responded particularly well to treatment and two patients who, although showed some improvement, responded less well. Multilevel modeling showed that the frequency of primary adaptive emotions (assertive anger, grief, and self-compassion) increased during the course of treatment for the good outcome cases but not for the poor outcome cases. The absolute frequency of primary adaptive emotions was significantly higher in the good-outcome cases compared to the poor-outcome cases. Overall, these findings provide initial support for the theory of change in EFT for SAD.

## 6. Conclusions and Future Directions

Although research on the effectiveness and mechanisms of EFT for SAD is at its initial phase, further developing EFT for this chronic and disabling disorder seems to be an important endeavor. Given that most patients with anxiety disorders do not receive evidence-based psychological treatment, having just cognitive-behavioral therapies as the only evidence-based treatment option for SAD is suboptimal. Although CBT for SAD is quite effective, many patients either do not have access to CBT or do not respond well to CBT, or simply prefer other forms of treatment (treatment preference is an important predictor of treatment success [103]). Having more than one empirically-based treatment option is important for theoretical reasons as well. Different treatments that work through different mechanisms provide more complete theoretical understanding regarding psychopathological processes responsible for the development and maintenance of the disorder. For example, if a treatment that is not based on cognitive reappraisal or exposure is effective, it suggests that SAD is not solely based on maladaptive beliefs or fear conditioning, and that we need to investigate additional etiological factors. It seems that there is a growing understanding that working directly with emotions is a critical ingredient in working with socially anxious patients. Emotion-focused work is now being incorporated into psychodynamic treatment [104] and cognitive therapy [105,106,107] for social anxiety disorder. A long tradition of research on exposure tells us that remaining at a cognitive-intellectual level is often not enough for change, and that patients need to feel their feelings in order to heal. However, EFT goes one step further: if exposure is designed to down-regulate symptomatic anxiety (via habituation or inhibitory learning), EFT aims to up-regulate shame, and then to upregulate primary adaptive emotions in order to transform shame.

Although research on EFT for SAD is at the very beginning, initial findings suggest that EFT has the potential to become an effective treatment option for SAD, and preliminary findings support its mechanism of change. It seems that the next phase of the treatment development research program of EFT should compare the efficacy of EFT and CBT in a randomized trial. Such a trial can serve as a platform for investigating treatment moderators and mechanisms of change [108]. By identifying moderator variables (i.e., baseline predictors of response to treatment), we will be able to identify which patients might benefit from which treatment and thus improve the overall number of responders. Thus far, studies aiming to identify baseline predictors of treatment outcome in SAD have largely produced inconclusive results [19,109]. By better understanding change mechanisms, we will be able to develop a deeper and more accurate understanding of what leads to change and develop and refine interventions accordingly, thus improving both treatments.

EFT directly addresses core psychopathological features of SAD, such as high levels of self-criticism, emotional avoidance, and trauma-related processes. In EFT, shame is viewed as a core underlying process in SAD, and a primary goal is to help patients attend to their shame, accept it, tolerate it, and make meaning of it. Using various techniques, patients are helped to activate shame so that it can be exposed to adaptive emotions, such as assertive anger that promotes boundary-setting, sadness of grief that promotes receiving comfort and soothing, and self-compassion. A primary assumption in EFT is that these adaptive emotions are healing factors, as they connect the patient to healthy adaptive needs, which in turn leads to empowerment that undoes the sense of inadequacy inherent in shame.
